# Reduction in C-terminal amidated species of recombinant monoclonal antibodies by genetic modification of CHO cells

**DOI:** 10.1186/1472-6750-14-76

**Published:** 2014-08-14

**Authors:** Mihaela Škulj, Dejan Pezdirec, Dominik Gaser, Marko Kreft, Robert Zorec

**Affiliations:** 1 Sandoz Biopharmaceuticals, Mengeš, Lek Pharmacetucals d.d, Kolodvorska 27, 1234 Mengeš, Slovenia; 2 Celica Biomedical Centre, Tehnološki Park 24, 1000 Ljubljana, Slovenia; 3 Laboratory of Neuroendocrinology – Molecular Cell Physiology, Institute of Pathophysiology, Faculty of Medicine, University of Ljubljana, Zaloška 4, 1000 Ljubljana, Slovenia; 4 Biotechnical Faculty, University of Ljubljana, Večna Pot 111, 1000 Ljubljana, Slovenia

**Keywords:** C-terminal amidation, Genetic modification, Recombinant monoclonal antibody

## Abstract

**Background:**

During development of recombinant monoclonal antibodies in Chinese hamster ovary (CHO) cells, C-terminal amidated species are observed. C-terminal amidation is catalysed by peptidylglycine α-amidating monooxygenase (PAM), an enzyme known to be expressed in CHO cells. The significant variations between clones during clone selection, and the relatively high content of amidated species (up to 15%) in comparison to reference material (4%), led us to develop a cell line with reduced production of C-terminal amidated monoclonal antibodies using genetic manipulation.

**Results:**

Initial target validation was performed using the RNA interference approach against *PAM*, which resulted in a CHO cell line with C-terminal amidation decreased to 3%. Due to the transient effects of small-interfering RNAs, and possible stability problems using short-hairpin RNAs, we knocked-down the *PAM* gene using zinc finger nucleases. Plasmid DNA and mRNA for zinc finger nucleases were used to generate a *PAM* knock-out, which resulted in two CHO cell lines with C-terminal amidation decreased to 6%, in CHO *Der2* and CHO *Der3* cells.

**Conclusion:**

Two genetically modified cell lines were generated using a zinc finger nuclease approach to decrease C-terminal amidation on recombinant monoclonal antibodies. These two cell lines now represent a pool from which the candidate clone with the highest comparability to the reference molecule can be selected, for production of high-quality and safe therapeutics.

## Background

The production of biopharmaceuticals for human use began in 1982 with recombinant insulin, and the development of new biopharmaceuticals has grew almost exponentially ever since. Over the past two decades, Chinese hamster ovary (CHO) cells have become the standard mammalian host cell line, with the expression and production of nearly 70% of all biopharmaceuticals [1,2]. CHO cells provide efficient post-translational modifications, which allow the production of recombinant proteins with glycoforms that are both compatible with, and bioactive in humans [1]. CHO cells can also be easily manipulated genetically, which has become of great importance more recently [3]. These two characteristics are especially important in the production of biosimilars, where achieving the correct extent of similarity to the reference molecule is a great challenge. The nucleotide sequence of the gene that encodes amino-acid sequence of the desired protein is the same as for the reference molecule. In contrast, post-translational modifications that are the consequence of metabolic pathways can differ between host cell lines, clones, cultivation conditions, medium composition, specific productivity and physiologic state of the cell [4]. Consequently, these need to be fine-tuned during development. In addition to posttranslational modifications, different charge variants can result in heterogeneity in the production of monoclonal antibodies (mAbs). These modifications potentially result in changes to the bioactivity, bioavailability or immunogenicity of the mAbs, and they therefore need to be additionally characterised to ensure the safety, quality and efficacy of the product. Among these, C-terminal amidated structures on the heavy chains of mAbs have recently attracted particular attention [5-8].

C-terminal α-amidation is catalysed by peptidylglycine α-amidating monooxygenase (PAM), and this protein modification is often required to confer full biological activity to peptide hormones [6,9-11]. Amidation is catalysed starting from a glycine-extended prohormone, by two sequential actions of two enzymes, peptidylglycine α-hydroxylating monooxygenase and peptydilamido-glycolat lyase. In mammals, both of these enzymes are derived from a single gene, which gives rise to the bi-functional PAM protein [12]. PAM thus catalyses the conversion of peptidylglycine substrates into α-amidated products in a two-step reaction, and it is the only enzyme known to catalyse the formation of amidated peptides [13].

Recently, large proteins like immunoglobulins have been reported to be substrates for PAM [6,7], and the expression of PAM in CHO cells was reported previously [14]. Tsubaki et al. reported that C-terminal α-amidation was detected in 8 of 12 recombinant mAbs, with ratios from 0.3% to 25.9% [6]. During our studies, we have also observed the presence of C-terminal amidated species in recombinant mAbs produced in CHO cells. Prolinamide was detected in up to 14% of all mAb molecules, which was too high to accomplish the desired similarity to the reference molecule.

It was previously shown that the level of mAb amidation in CHO cells can be affected via bioprocesses and medium optimisation, with the addition of copper to the culture medium [7]. On the other hand, metabolic engineering is becoming a powerful tool to manipulate expression hosts for improved product quality, and the introduction of a PAM knocked-down cell line that can produce mAbs with desired comparability to a reference molecule would be a state-of-the-art solution.

In the present study, the expression of PAM, and consequently the C-terminal amidation of recombinant mAbs, was reduced by two approaches: gene manipulation using RNA interference (RNAi) and zinc finger nucleases (ZFN). RNAi has been efficiently used for down-regulation of desired genes, and it can be performed using chemically synthesised small-interfering (si)RNA molecules, or via the endogenous expression of short-hairpin (sh)RNA molecules that are encoded by plasmid vectors [15,16]. While siRNAs can provide transient knock-down of expression of a target gene, shRNA vectors can induce long-term expression of RNAi silencing in a target cell [15]. The ease and rapidity of these RNAi approaches have made them the method of choice for initial target validation.

However, using RNAi, complete elimination of expression of the target gene is rarely obtained. To achieve permanent gene knock-out, ZFNs can offer a distinct advantage. Moreover, the growing number of reports using ZFNs across different species suggests that ZFN-mediated gene disruption is a robust and general method for targeted gene knock-out. As this approach results in an alteration of the genome itself, these mutations are transmitted stably through all of the subsequent generations of the cell line, as is the case with conventional gene targeting [3,17]. Rapid gene knock-out can be achieved by using a ZFN approach to create a double-stranded break in the locus of interest, which allows it to be repaired by non-homologous end-joining; this ligates the two broken ends, with the occasional loss of genetic information. ZFNs can therefore be used to introduce small deletions at the site of such a break, an outcome that can be exploited to disrupt a target gene [18,19]. The high frequencies of gene disruption strongly support the likelihood of achieving a desired genotype, in which each copy of the target gene is functionally knocked out in 1% to 50% of all cells [17,18].

## Results and discussion

### siRNA and shRNA experiments

C-terminal amidation is a common posttranslational modification that is seen on therapeutic mAbs [6], and which has also been observed during recombinant mAb development (data not shown). The significant variations between clones during clone selection, and the relatively high content of amidated species in comparison to reference molecules, directed us towards the development of a cell line with reduced production of C-terminal amidated species.

RNAi has already been efficiently used for the improvement of cellular productivity and the quality of recombinant proteins produced in CHO cells. RNAi had thus been used for silencing apoptosis-associated gene expression [20-23], glycosylation-associated gene expression [24-26], and gene expression of lactate dehydrogenase [27] and dihydrofolate reductase [28,29].

**Figure 1:**
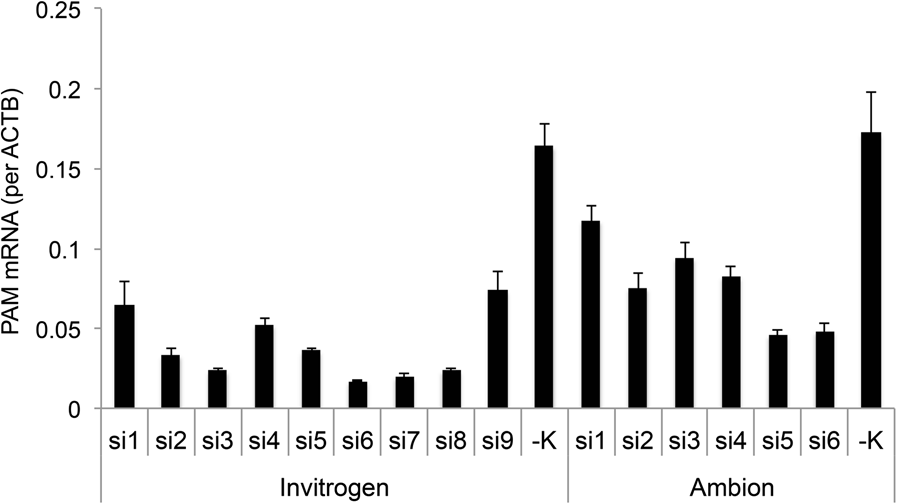
**Silencing of *PAM *using siRNA.***PAM* mRNA expression level after transfections using the siRNAs (Invitrogen, Ambion, as indicated) in the CHO *Der2* parental cell line*.* In comparison to the negative control (−K), there was up to 8-fold reduction in the mRNA expression levels using the si6 Invitrogen siRNA, and up to 5-fold reductions using the si5 and si6 Ambion siRNAs. Overall, better silencing effects were obtained using the Invitrogen siRNAs. The mRNA expression levels were determined using qPCR (calculated per *ACTB* housekeeping gene), and the data are means ± standard deviations of the two biological replicates.

**Figure 2:**
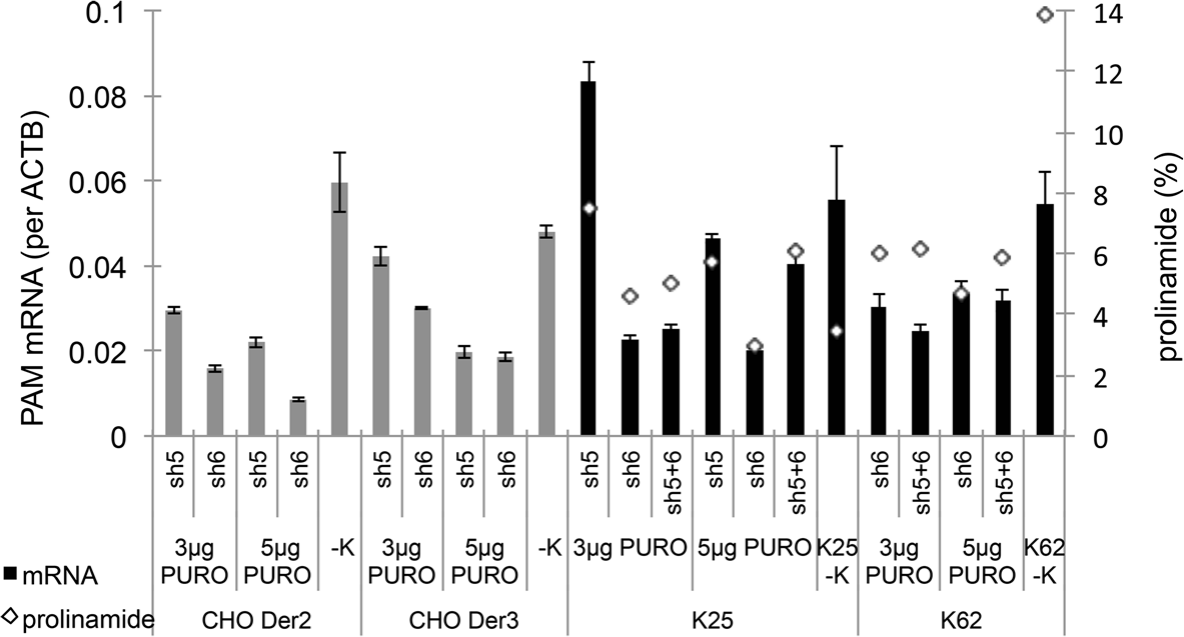
**Silencing of *PAM *using shRNA.** The mRNA expression levels are shown after the transfections using the Ambion sh5 and sh6 shRNAs on the CHO *Der2* and CHO *Der3* parental cell lines and on the K25 and K62 clones derived from the CHO *Der3* parental cell line. The data obtained for the parental cell lines are in grey. In comparison to the negative controls (CHO *Der2* -K, and CHO *Der3* -K), the highest silencing effects were achieved with shRNA sh6 and 5 μg/ml antibiotic selection (5 μg puromycin [PURO]). Up to 3.7-fold reduction in the mRNA expression levels was seen for the CHO *Der2* cell line, and up to 2.6-fold reduction for the CHO *Der3* cell line. The data for the mAb-expressing clones K25 and K62 are in black. In this case, the *PAM* expression level and the prolinamide content (%) are presented. In comparison to the negative controls (K25 -K, and K62 -K), the different antibiotic selections did not show any differences in mRNA expression levels and prolinamide content. Up to 3.5-fold reduction in mRNA expression levels was observed for K25, and up to 2.2-fold reduction in K62. Prolinamide was decreased from 3.5% to 3% for K25, and from 14% to 4.6% for K62, which represents a 3-fold decrease. The mRNA expression levels for parental cell lines and the K25 and K62 clones were determined using qPCR (calculated per *ACTB* housekeeping gene), and the data are means ± standard deviations of two biological replicates.

Fifteen different siRNAs were designed on the basis of the *C. griseus PAM* nucleotide sequence and tested on the CHO *Der2* parental cell line. Up to an 8-fold decrease was observed in PAM mRNA expression levels using siRNAs from Invitrogen, and up to a 5-fold decrease using siRNAs from Ambion (Figure 1). On the basis of these data, and due to shRNA design limitations, siRNAs si5 and si6 (Ambion) were selected for the design of shRNAs, to obtain long-term silencing of *PAM*. siRNA knock-down of the target lasts for 3 to 5 days, and therefore to induce long-term expression of RNAi silencing in the target cells, an shRNA vector has to be used [15]. The shRNA silencing effect was tested on two different CHO parental cell lines, CHO *Der2* and CHO *Der3*, and on two mAb-expressing clones derived from the CHO *Der3* cell line, clone K62 with high (14%), and clone K25 with low (4%), prolinamide contents in the mAb that was produced (Figure 2). After shRNA transfection and antibiotic selection, all of the generated pools were analysed by cation-exchange chromatography (CEX), for evaluation of the prolinamide content (Figure 2). The shRNA designed on the basis of the si6 siRNA was shown to have the most potent silencing effect on all of the transfected cell lines (Figure 2).

The data presented in Figure 2 show the correlation between *PAM* mRNA expression levels and C-terminal amidation of the recombinant mAb. Up to a 4-fold decrease in mRNA and a 3-fold decrease in prolinamide content were observed. As can be seen from Figure 2, the prolinamide content for clone K62 was decreased to 4.6%, which represents a 3-fold decrease, and for clone K25, where the initial starting point was 3.5% prolinamide content, only a minimal reduction was obtained. Nevertheless, there is an interesting observation here that should be considered. No matter which clone is considered, as one with a previously high (14%) or low (4%) prolinamide content, the reduction in the prolinamide content after shRNA knock-down never decreased below 4%, which is the same as the level of the reference molecule.

### ZFN experiments

The shRNA experiments gave very promising results, as they yielded PAM levels that were comparable to the reference molecule. However, possible toxicity effects on long-term expression and the additional metabolic load on the cells due to the overexpression of these factors during times of stress might also influence cell performance. In addition, shRNA-mediated knock-down relies on the constant expression of repressor molecules, which can be unstable in the knocked-down cells in the long term [17]. RNAi instability was reported by Lim et al. in the silencing of the *BAX* and *BAK* genes, where 1% of the screened clones was silenced for both genes, but there was instability of the generated knock-down clones [20]. This is an undesirable feature during the mAb production process, as it can cause heterogeneity of the product [20]. To avoid such problems, rapid and permanent gene knock-out using ZFNs can offer distinct advantages. Moreover, the growing number of reports using ZFNs across different species have suggested that ZFN-mediated gene disruption is a robust and general method for targeted gene knock-out. ZFN-mediated gene knock-out requires only transient expression of the ZFNs, and it can result in a permanent genetic mutation that is stably transmitted through all of the subsequent generations of the cell line [17].

The first reported example of the use of engineered ZFNs to disrupt an endogenous locus in a mammalian cell was a knock-out of the dihydrofolate reductase gene in CHO cells. The observed bi-allelic mutation rate was 2% to 3%. In comparison to traditional methods, this frequency of gene disruption is very high, and it increases the possibility of achieving the desired genotype in which each copy of the target gene is functionally knocked out [17].

ZFNs have also already been successfully used for the generation of many different knocked-out CHO cell lines. They have been reported as used to create triple knock-out CHO cells, with the disruption of the two selectable marker genes of glutamine synthetase and dihydrofolate reductase, as well as the gene encoding α-1,6-fucosyltransferase (FUT8) [30]. *BAK* and *BAX* deletion was also achieved, to produce apoptosis-resistant CHO cells [31]. Additionally a *FUT8* knock-out CHO cell line has been reported [32,33].

Thus, ZFNs were used to create a cell line with stably knocked-down *PAM* expression, so as to reduce C-terminal amidated species on the target mAb. These ZFNs were designed on the basis of a sequence that had been shown to generate silencing effects using RNAi. Initially, ZFN plasmid DNA was used for the transfections of the parental CHO *Der2* and CHO *Der3* cell lines. The knock-out effect was evaluated by determination of the *PAM* gene copy number using qPCR. This method was evaluated using the Cell-I Nuclease Mismatch assay (data not shown), which is usually used to evaluate knock-out clones. The qPCR method has been shown to be less laborious and time consuming, and therefore it enables efficient screening of the high numbers of clones generated.

**Figure 3:**
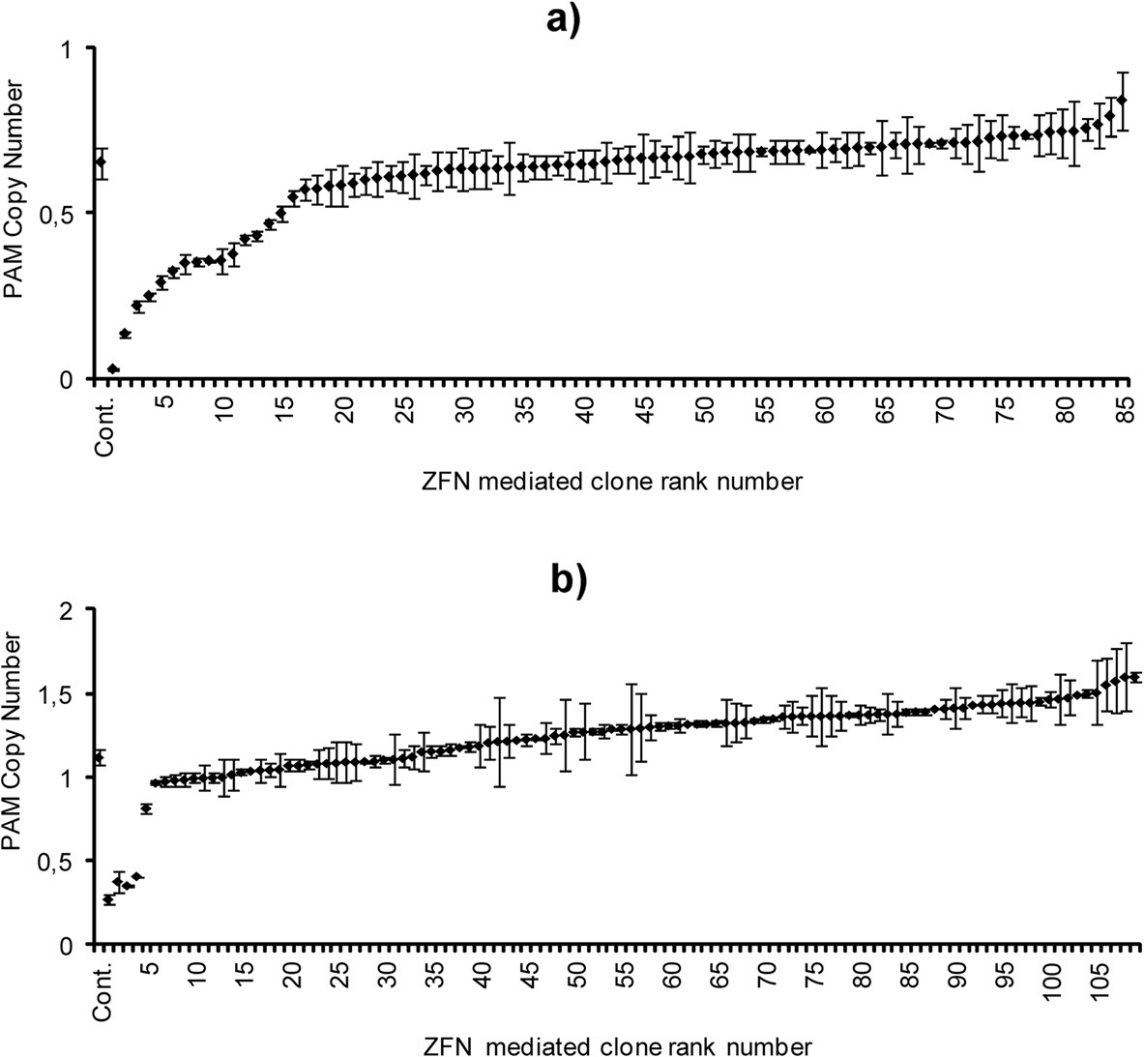
***PAM *gene copy numbers for the clones derived from the CHO *Der2 *and CHO *Der3 *cell lines transfected using the ZFN plasmid DNA.** The PAM gene copy numbers for the 85 ZFN clones derived from the CHO *Der2* parental cells **(a)** and the 109 ZFN clones derived from the CHO *Der3* parental cells **(b)**, transfected using the ZFN plasmid DNA. The copy numbers were determined using qPCR, and the data are means ± standard deviations of two technical replicates.

In all, 85 clones were generated from the CHO *Der2* cell line, and 109 clones from the CHO *Der3* cell line. In contrast to the CHO *Der2* cell line, which is diploid, the CHO *Der3* cell line was shown by karyotyping to be a triploid (data not shown), and a knock-out was therefore more difficult to achieve. A partial reduction of the *PAM* copies in the CHO *Der2* and CHO *Der3* cell lines was achieved (Figure 3). Reported knock-out efficiencies for single copy genes have been higher than 1% [17,30,34], and have reached 5% [33]. Thus, to detect a single clone with a total knock-out that would be reflected in a mAb without functional *PAM*, around 100 clones would need to be generated, and in the case of the triploid CHO *Der3* cell line, this would be higher.

The *PAM* mRNA expression was evaluated for the 10 best clones by qPCR, and high correlation was observed with the copy number determination at the genomic DNA level (data not shown).

To determine the mRNA expression levels, the *PAM* mRNA was calculated per housekeeping gene. The choice of a housekeeping gene can often be quite challenging, as expression is usually not the same across different CHO cell lines and clones, and can also vary according to the physiological state of the cells. To avoid these effects, four different housekeeping genes were tested in the present study: *ACTB*, *GAPDH*, *G6PD* and *EF1* (Table 1). All four of these housekeeping genes were shown to be suitable for the calculation of the mRNA expression levels (data not shown), with the data below are based on the use of *ACTB* as the housekeeping gene.

**Table 1:** Nucleotide sequences of the qPCR primers and probes used in the present study

	**Forward primer**	**Reverse primer**	**Probe**
PAM	GGCCGGATCCAATGTTTCAGAA	TCCCAAATGACGCATGTTTAATCTCT	CTGACACCAAAGAATTT
ACTB	AGCCACGCTCGGTCAG	CATCCTGCGTCTGGACCT	CCGGGACCTGACAGACT
GAPDH	CTGATTTTTCTTGCGTCGAGTTT	GAGTTGTGTTTGTGGACGAAGTAC	TCCGGTAAGACCTTTCG
EF1	CCTGGAATGGTGGTTACCTTTG	CATGGTGCATTTCAACAGACTTTACT	CCAGTCAACGTTACAACAGA
G6PD	ACAACATTGCCTGTGTGATCCT	CCCAAATTCATCAAAGTAGCCC	CCACGACCCTCAGTACCA

PAM, peptidylglycine amidating monooxygenase; ACTB, β-actin; GAPDH, glyceraldehyde-3-phosphate dehydrogenase; EF1, elongation factor 1; G6PD, Glucose-6-phosphate dehydrogenase.

**Figure 4:**
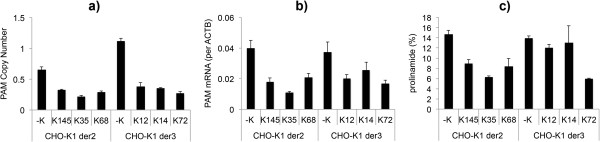
***PAM *gene copy numbers, mRNA expression and C-terminal amidation for clones transfected using ZFN pDNA.** The *PAM* gene copy numbers **(a)**, *PAM* mRNA expression levels (**b**; calculated per *ACTB* housekeeping gene), and prolinamide content on the recombinant mAb **(c)** for the three best ZFN knock-out clones derived from the CHO *Der2* and CHO *Der3* parental cell lines, where ZFN pDNA was used for the transfection. Prolinamide was reduced to 6%. The copy numbers and mRNA expression levels were determined using qPCR, and the data are means ± standard deviations of two technical replicates. The prolinamide content was determined using CEX, and these data are means ± standard deviations of two biological replicates.

The three best clones were transfected with a plasmid containing the mAb expression cassette. After 4 weeks of antibiotic selection and 14 days of fed-batch cultivation, the mAbs produced from these three clones were evaluated for the presence of prolinamide, using CEX. The data from the *PAM* gene copy numbers, mRNA expression levels, and prolinamide content are presented in Figure 4. The prolinamide levels for both of the CHO *Der2* and CHO *Der3* cell lines after evaluation using CEX were determined to have decreased to 6%. As observed with the shRNA (see above), there was correlation between copy number, mRNA expression, and prolinamide content of the recombinant mAb (Figure 4).

Transfections using ZFN plasmid DNA result in its random integration into the genome of CHO cells and continuous expression of the ZFN. Genome integration is not desired, due to the continuous ZFN activity, and thus potential off-target effects. The mechanism of ZFN target recognition has been reported to be highly specific, with usually an 18-bp site recognized and the dimerisation of two ZFNs needed to cause a double-stranded break [18,35]. Despite this, some off-target effects have also been observed. Off-target effects of 5.4% were reported for the disruption of the CCR5 HIV receptor by Klug et al. [19].

To avoid this, the transfections were performed using ZFN mRNA, for transient ZFN expression. The ZFN mRNA was prepared in-house using *in-vitro* transcription from the two ZFN plasmid DNAs. The cells were then transfected with the two ZFN mRNAs; i.e., ZFN1 and ZFN2. Various amounts for the combination of these two mRNAs were tested: total mRNAs of 30 μg to 60 μg per transfection. The 50 μg mRNAs transfection (25 μg ZFN1 mRNA, 25 μg ZFN2 mRNA) was the most promising combination. The concentrations of the mRNAs used for the transfections correlated with the silencing effects, although the higher mRNA concentrations decreased the cell viability to <80% (data not shown). The pools were evaluated by qPCR on day 4 following transfection. A 50% knock-out was achieved for the generated pools, and the cell viability was >80%, which is needed for seeding into the semi-solid medium for clone generation using ClonePix.

**Figure 5:**
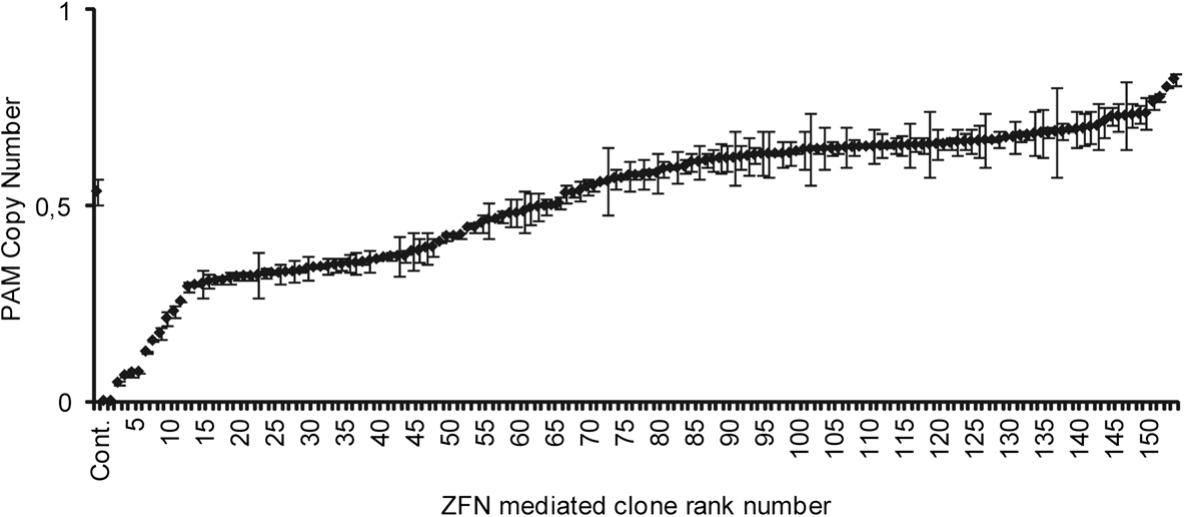
***PAM *gene copy numbers for 154 ZFN clones from CHO *Der2*parental cell line transfected using ZFN mRNA.** The two clones (K62 and K68) identified as knocked-out for all *PAM* gene copies at the genomic DNA level. The copy number was determined by qPCR, and the data are means ± standard deviations of two technical replicates.

**Figure 6:**
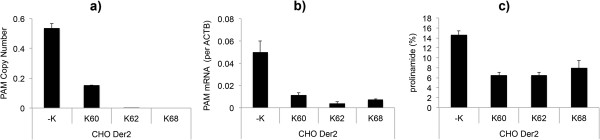
***PAM *gene copy numbers, mRNA expression, and C-terminal amidation for the clones transfected using ZFN mRNA.** The *PAM* gene copy numbers **(a)**, *PAM* mRNA expression levels (**b**; calculated per *ACTB* housekeeping gene), and prolinamide contents of the recombinant mAb determined by CEX **(c)**, for the three best ZFN knock-out clones derived from the CHO *Der2* parental cell line, where ZFN mRNA was used for the transfection. The two clones identified (K62 and K68) were knocked-out for all *PAM* gene copies at the genomic DNA level, with mRNA expression and a prolinamide content of 6% still observed. The copy numbers and the mRNA expression levels were determined using qPCR, and the data are means ± standard deviations of two technical replicates. Prolinamide content was determined by CEX, results are presented as an average of two biological replicates.

These mRNA transfections were used only with the CHO *Der2* cell line. Here, 154 clones were evaluated and two were determined to be completely knocked-out at the genomic DNA level by qPCR (Figure 5), which represented a 1% knock-out efficiency. The 10 best clones were again tested for *PAM* mRNA expression (data not shown), and afterwards the best three of these 10 clones were transfected with a plasmid containing the mAb expression cassette, in duplicates, and evaluated by CEX (using the same procedure as described for the plasmid DNA transfections). These data are presented in Figure 6.

The C-terminal amidated structures in all of these tested clones decreased to a limit of 6%, which was the same as observed using ZFN plasmid DNA. mRNA expression and C-terminal amidation were observed for all of these tested clones, even though this was not expected for clones K62 and K68.

Down-regulation of *PAM* did not have any influence on cell growth, in comparison to the non-silenced controls. All of the cells showed similar cell growth, which did not differ from that expected for CHO cells. Additionally, there was no correlation between cell productivity and C-terminal amidation of the produced mAb (data not shown).

The silencing effects that were obtained using the RNAi approach showed more promising results in terms of prolinamide reduction on the recombinant mAb. In this case, prolinamide was reduced to 3%. As indicated above, siRNA effects are only transient [15] and the shRNA approach is also questionable, due to long-term stability problems and the additional metabolic burden on the cells. Using the ZFN approach, the decrease in prolinamide was not as effective as with shRNA, as it was reduced to 6%. On the other hand, this ZFN approach has the advantage of producing a stable genetic modification, which is very important for the production scale.

After transfection with the mAb-expressing plasmid, each of these two generated ZFN knocked-down cell lines (CHO *Der2* and CHO *Der3* cells) represented a mixed cell population with a mean prolinamide content of 6%. In comparison to the negative controls, which generated 15% (CHO *Der2*) and 14% (CHO *Der3*) prolinamide content, this represents a decrease in the prolinamide content of >50%. These pools with mean prolinamide content of 6% thus represent good starting points for the isolation of candidate clones that express different amounts of C-terminal amidated species, thus providing the choice for the one that is the most comparable to the reference molecule.

The higher efficiency of the knock-down achieved using shRNA in comparison to ZFN might be because the shRNA targets the degradation of the *PAM* mRNA, which will reduce its expression overall. For the ZFNs, the knock-out effects are at the level of the genomic DNA, and if total knock-out is not achieved in all of the copies, the expression of a functional mRNA will still take place, and thus the translation into the functional PAM protein will not be fully blocked. Disruption of the target gene can occur by causing a shift in the reading frame, the generation of a premature termination codon, or the deletion of critical amino-acid residues. However, the result of non-homologous end joining repair might be only an amino-acid point mutation or a deletion of the protein molecule, which might result in a partially or fully functional enzyme.

However, the goal of the present study was not to generate a completely knocked-out CHO cell line, but to decrease the content of the C-terminal amidated structures to a level that is sufficiently comparable to the reference molecule, and this was successfully achieved using both approaches.

## Conclusions

During development, C-terminal amidated species are detected on recombinant mAbs, an observation that was also recently reported in other studies [6]. C-terminal amidation can be reduced by development and optimisation of the grwth medium, and use of cofactors such as copper [7], although these approaches are project specific and time consuming. Due to its extensive development, metabolic engineering has provided a powerful tool to diminish the expression of the *PAM* gene through gene alterations using RNAi and ZFNs. In the present study, both of these approaches were tested. The RNAi was conducted for the initial target validation, for the later ZFN knock-out. Using the shRNA approach, decreases in C-terminal amidation were achieved for two mAb-producing clones: the K25 with low prolinamide content (4%), and the K62 clone with high (14%) prolinamide content, which were decreased to 3.5% and 5%, respectively. The prolinamide levels of the reference molecule were 4%, and therefore a comparability level was successfully achieved. In this case, the gene silencing was performed on two clones that were already expressing the desired mAb.

For recombinant mAb production, a parental cell line with stably reduced *PAM* expression is desired. To achieve this, genome alterations using ZFNs are the more appropriate tool, as breaks are created at the genomic DNA level that are stably transferred through the subsequent generations of the cell line [3,17]. Two CHO cell lines were generated, as CHO *Der2* and CHO *Der3* cells, and they were evaluated for *PAM* at the genomic DNA and mRNA expression levels. After the selection of the three most promising clones, these were transfected with a mAb-producing plasmid and evaluated for the C-terminal amidated species. The C-terminal amidation was decreased to 6% in both of these cell lines, which represents a 50% lower content in comparison to the ZFN non-treated controls. The two generated cell lines now represent two pools from which candidate clones with the highest comparability to the reference molecule can be selected.

Thus, this use of genetically manipulated cell lines now gives us the opportunity to go even further towards providing high-quality and safe therapeutics.

## Methods

### Design of siRNAs, shRNAs and ZFNs

The Chinese hamster *(Cricetulus griseus) PAM* nucleotide sequence was derived from a public database (Table 2). The siRNAs, shRNAs and ZFNs were constructed on the basis of this sequence.

**Table 2:** **The *Cricetulus griseus PAM *** **gene sequence used for the RNAi and ZFN construction**

***PAM*** **gene nucleotide sequence**	GGGAGTGCTCCTAAGCCAGGCCAGTTCAGTGTTCCTCACAGTTTGGCCCTTGTGCCTCATTTGGACCAGTTGTGTGTGGCAGACAGGGAAAATGGCCGGATCCAATGTTTCAGAACTGACACCAAAGAATTTGTGAGAGAGATTAAACATGCGTCATTTGGGAGAAATGTATTCGCAATTTCATATATATCAGGTTTGCTCTTTGCAGTAAATGGGAAGCCTTACTTTGGAGACCATGAACCTGTGCAAGGCTTTGTGATGAACTTTTCCAGTGGGGAAATTATAGATGTCTTCAAGCCAGTACGCAAGCACTTTGACATGCCTCACGATGTGGTTGCCTCTGACGATGGGAATGTGTACATTGGAGACGCACACACGAACACGGTGTGGAAGTTCACCCTGACTGAAAAAATGGAGCATCGATCGGTTAAAAAGGCAGGCATTGAGGCTCAGGAAATCAAAGAAACCGAGGCAGTTGTTGAATCCAAAATGGAGAACAAACCCACCTCCTCAGAATTGCAGAAGATGCAAGAGAAACAGAAACTGATCAAAGAGCCAGGTTCGGGAGTGCCCGTGGTTCTCATTACAACCCTTCTGGTTATTCCTGTGGTTGTCCTGCTGGCCATTGTCATGTTTATTCGGTGGAAAAAATCAAGGGCCTTTGGAGGAAAA

The online design tools of Invitrogen and Ambion were used to design the siRNA sequences (nine according to Invitrogen, six according to Ambion; Table 3). After the siRNA evaluation, shRNAs were designed using the Ambion online design tool. Two complementary oligonucleotides were synthesised for each shRNA by Methabion (Table 4), and then these were annealed in-house, to generate the double-stranded oligonucleotides. Subsequently, these annealed oligonucleotides were cloned into the *pSilencer* 2.1-U6 puro vector (Ambion). DNA sequencing was performed to verify the sequences of the oligonucleotide inserts.

**Table 3:** Nucleotide sequences of the siRNAs used in the present study

**Name**	**siRNA oligonucleotide**	**Supplier**
siRNA_si1	CAGUUGUGUGUGGCAGACAGGGAAA	Invitrogen
siRNA_si2	CGGAUCCAAUGUUUCAGAACUGACA	Invitrogen
siRNA_si3	CCAAUGUUUCAGAACUGACACCAAA	Invitrogen
siRNA_si4	GAGAGAGAUUAAACAUGCGUCAUUU	Invitrogen
siRNA_si5	CAUGCGUCAUUUGGGAGAAAUGUAU	Invitrogen
siRNA_si6	UGGGAGAAAUGUAUUCGCAAUUUCA	Invitrogen
siRNA_si7	GGGAGAAAUGUAUUCGCAAUUUCAU	Invitrogen
siRNA_si8	CACACGAACACGGUGUGGAAGUUCA	Invitrogen
siRNA_si9	CAAAGAAACCGAGGCAGUUGUUGAA	Invitrogen
siRNA_si1	CAGUAAAUGGGAAGCCUUATT	Ambion
siRNA_si2	AGGCAGUUGUUGAAUCCAATT	Ambion
siRNA_si3	AACAGAAACUGAUCAAAGATT	Ambion
siRNA_si4	CAAGAGAAACAGAAACUGATT	Ambion
siRNA_si5	GAACUGACACCAAAGAAUUTT	Ambion
siRNA_si6	UUUCAGAACUGACACCAAATT	Ambion

**Table 4:** Nucleotide sequences of the shRNAs used in the present study

**Name**	**shRNA top strand oligonucleotide**	**shRNA bottom strand oligonucleotide**
shRNA_sh5	GATCCGAACTGACACCAAAGAATTCTCAAGAGAAATTCTTTGGTGTCAGTTCTGTTTTTTGGAAA	AGCTTTTCCAAAAAACAGAACTGACACCAAAGAATTTCTCTTGAGAATTCTTTGGTGTCAGTTCG
shRNA_sh6	GATCCGTTTCAGAACTGACACCAAACTCAAGAGATTTGGTGTCAGTTCTGAAACATTTTTTGGAAA	AGCTTTTCCAAAAAATGTTTCAGAACTGACACCAAATCTCTTGAGTTTGGTGTCAGTTCTGAAACG

For the ZFN experiments, two ZFN plasmids that targeted the *PAM* gene (pZFN PAM1, pZFN PAM2) were designed by Sigma (CompoZr^TM^ Custom Zinc Finger Nuclease, PN. SAFCZFN-1kt). These two ZFN plasmids were later used as templates for the preparation of PAM1 and PAM2 mRNA, by *in-vitro* transcription using MessageMax T7 ARCA-Capped Transcription Kit (Cellscript). The transcripts were poly-A tailed using A-Plus Poly(A) Polymerase Tailing Kit (Cellscript), and purified using RNeasy Micro Kit (Qiagen). The RNA concentrations were determined by the NanoDrop, and the transcript sizes were evaluated using a Bioanalyser.

### Cell culture and transfection

#### siRNA and shRNA experiments

Two different CHO parental cell lines (CHO *Der2* and CHO *Der3*) and two different mAb-producing clones that were derived from the CHO *Der3* cell line (clone K25 and clone K62) were used in this study. Clones K25 and K62 were prepared by nucleofection of the parental CHO *Der3* cell line using a plasmid vector containing the mAb expression cassette. The cells were cultivated at 37°C, under 10% CO_2_, and with agitation at 90–110 rpm, in animal-component-free growth medium. The C-terminal amidation of the mAb in clone K25 was determined as 4%, and in clone K62, as 14%, using CEX. It was shown that 4% of all of the reference molecules contained C-terminal amidated structures.

For the siRNA experiments, CHO *Der2* cells were transfected with siRNAs, in duplicate, using nucleofection, and cultivated for 4 days. On day 4, the cell pellets were collected for analysis of gene expression by qPCR.

For the shRNA experiments, all four cell lines (CHO *Der2*, CHO *Der3*, clone K25, and clone K62) were transfected with shRNAs, in duplicate, using nucleofection. Antibiotic selection of all of the transfected pools was performed using 3 μg/ml puromycin. The puromycin was added to the cell culture 2 days after the transfection, when the cell viability exceeded 60%. The cells were split on a 2-2-3-day schedule at 2-3E5 cells per ml, in the appropriate pre-warmed medium to maintain exponential cell growth. After reaching the appropriate cell density and viability, the cell pellets were collected for gene expression analysis by qPCR, and a 10-day simple batch containing 3 μg/ml puromycin was inoculated (after 10 days, the supernatants were collected, purified on protein A columns, and evaluated for prolinamide content using CEX). The cells were further cultivated in medium containing 5 μg/ml puromycin. After reaching the appropriate cell density and viability, this procedure was repeated.

#### ZFN experiments

For the ZFN experiments, the CHO *Der2* and CHO *Der3* parental cell lines were transfected using 3 μg of each ZFN plasmid (PAM1, PAM2), by nucleofection. After 4 days, the cells were seeded into the semi-solid medium, and on day 10 the colonies were transferred into 96-well plates by ClonePix FL. After colony formation in the 96-well plates, the individual colonies were transferred into 24-well plates, and later into 6-well plates. Finally, the clones were transferred into 125-ml shaking flasks, and after achieving the appropriate cell density and viability, the cell pellets were collected for the determination of gene copy number and gene expression by qPCR. The same procedure was repeated on the CHO *Der2* cell line using 25 μg of each ZFN mRNA (PAM1, PAM2), which was prepared in-house from the PAM1 and PAM2 ZFN plasmids.

After the selection of the three clones with the highest knock-down efficiency generated using ZFN plasmid DNA and mRNA, the transfections were performed using 3 μg mAb-expressing vector, in duplicate. After 4 weeks of antibiotic selection with geneticin, a 14-day fed-batch was inoculated. After 14 days, the supernatants were collected and purified using protein A columns. The prolinamide contents were evaluated using CEX.

### DNA/RNA purification and qPCR template preparation

The genomic DNA and the total RNA were isolated from pellets containing 5E6 cells on an automated QIAcube workstation. The genomic DNA was isolated using Blood & Cell Culture DNA Mini Kit (Qiagen), and the total RNA using RNeasy Mini Kit (Qiagen). After isolation, the nucleic acid concentrations were measured using the NanoDrop. After the determination of the total RNA concentrations, DNase I (Ambion) was added to 5 μg total RNA and incubated according to the manufacturer instructions. After this DNase treatment, the RNA was transcribed into cDNA using SuperScript VILO Kit (Invitrogen).

The genomic DNA and cDNA were used later as templates for the qPCR. The cDNA was used for evaluation of the siRNA and shRNA silencing effects, while the genomic DNA was used for evaluation of the knock-out effects of clones produced using the ZFNs.

### qPCR experiments

#### siRNA and shRNA experiments

The silencing effects of the siRNAs and shRNAs were examined by qPCR, using TaqMan chemistry and absolute quantification. The standard curve was constructed from CHO *Der1* genomic DNA, which was amplified using the *PAM* and *ACTB* primers (Table 1). The *PAM* and *ACTB* mRNA copy numbers for each sample were extrapolated from the standard curve, and the *PAM* mRNA expression levels were expressed as the number of *PAM* mRNA transcripts per reference *ACTB* gene. The standard curves were constructed from five dilution points, each as three parallel determinations. The *PAM* mRNA expression levels were determined as four dilution points, each as two parallel determinations for each sample.

#### ZFN experiments

The efficiencies of the ZFNs were determined by qPCR, using TaqMan chemistry and absolute quantification. The TaqMan probe was designed on the ZFN cutting site, and therefore no amplification of the *PAM* gene was expected in completely knocked-out clones. The standard curve was constructed from the CHO *Der1* genomic DNA amplified using the *GLUC* and *PAM* primers (Table 5). The copy numbers for both of these genes were extrapolated from the standard curve for each sample, and the ratio between them was calculated. *GLUC* is a single-copy gene in CHO cells, and was used as the reference gene for the *PAM* gene copy number determination. Standard curves were constructed from five dilution points, each as three parallel determinations. The *PAM* copy numbers were determined as two dilution points, each as three parallel determinations for each sample.

**Table 5:** Nucleotide sequences of the qPCR primers and probes used in the present study

	**Forward primer**	**Reverse primer**	**Probe**
**GLUC**	ATTGCCAAACGCCACGAT	CCAAGCAATGAATTCCTTTGC	CTGAAGGGACCTTTACCA
**PAM**	GCCCCAGCTCGGACATG	CCTTGCAGGGCGAGCA	CCGGCTGCTGCTGCT

GLUC, glucagon; PAM, peptidylglycine amidating monooxygenase.

After the selection of the 10 clones with the highest knock-out efficiency, the *PAM* expression was determined as described above, using four different housekeeping genes: *GAPDH*, *ACTB*, *G6PD* and *EF1* (Table 1).

### Cation-exchange chromatography

The mAb concentrations were determined using the ForteBio system. The mAbs were purified using protein A columns and analysed using CEX, with an analytical HPLC chromatographic system. Using this method, Lys and prolinamide were eluted in the same peak. The amount of prolinamide was then determined by C-terminus treatment with carboxypeptidase. Following the further analysis using the same CEX system, the remaining peak defined the prolinamide levels. The prolinamide levels are given as percentages of the C-terminal amidated species of the total mAb content.

After carboxipeptidase treatment, in addition to prolinamide, the basic peak can include multiple species, like N-terminal cyclised species, succinimide species, hybrid glycan-containing species, and others. Therefore the CEX analysis was always performed in parallel with the non-silenced and non-knockout controls, represented by samples from the same cell lines that had undergone blank transfections and were grown under the same conditions. The effects of RNAi and the ZFNs on the treated samples are highly specific, and the probability of other post-translational modifications other than reductions in prolinamide is very low. The reductions in the C-terminal amidation were determined relative to the controls, and therefore the reduction in the basic peak defined the reduction in C-terminal amidation.

## Competing interests

This study was performed at Sandoz Biopharmaceuticals, Mengeš, a Member of the Novartis group, which also financed the article-processing charges. The patent application entitled “Reduction of formation of amidated amino acids in cell lines for protein expression” (No. 12164264.9 - 1212) was filed in 2012. The authors confirm that there are no known conflicts of interest associated with this publication, and there has been no significant financial support for this study that might have influenced its outcome. The authors confirm that they have given due consideration to the protection of the intellectual property associated with this study, and that there are no impediments to its publication, including the timing of the publication, with respect to the intellectual property. In so doing, the authors confirm that they have followed the regulations of their institutions concerning intellectual property. The authors also confirm that the manuscript has been read and approved by all of the named authors, and that there are no other persons who satisfy the criteria for authorship who are not listed. All of the authors declare they have no competing interests of financial or non-financial natures.

## Authors’ contributions

MS and DP carried out all of the experiments and MS drafted the manuscript. DG participated in the planning the design of the study. DG, MK and RZ critically revised the manuscript and all authors approved the final version for publication.

## References

[B1] Kim JY Kim YG Lee GM CHO cells in biotechnology for production of recombinant proteins: current state and further potential Appl Microbiol Biotechnol 2012 93 917 930 22159888 10.1007/s00253-011-3758-5

[B2] Butler M Animal cell cultures: recent achievements and perspectives in the production of biopharmaceuticals Appl Microbiol Biotechnol 2005 68 283 291 15834715 10.1007/s00253-005-1980-8

[B3] Warner TG Enhancing therapeutic glycoprotein production in Chinese hamster ovary cells by metabolic engineering endogenous gene control with antisense DNA and gene targeting Glycobiology 1999 9 841 850 10460826 10.1093/glycob/9.9.841

[B4] Werner RG Kopp K Schlueter M Glycosylation of therapeutic proteins in different production systems Acta Paediatr Suppl 2007 96 17 22 17391433 10.1111/j.1651-2227.2007.00199.x

[B5] Harris RJ Processing of C-terminal lysine and arginine residues of proteins isolated from mammalian cell culture J Chromatogr A 1995 705 129 134 7620566 10.1016/0021-9673(94)01255-d

[B6] Tsubaki M Terashima I Kamata K Koga A C-terminal modification of monoclonal antibody drugs: amidated species as a general product-related substance Int J Biol Macromol 2013 52 139 147 23022270 10.1016/j.ijbiomac.2012.09.016

[B7] Kaschak T Boyd D Lu F Derfus G Kluck B Nogal B Emery C Summers C Zheng K Bayer R Amanullah A Yan B Characterization of the basic charge variants of a human IgG1: effect of copper concentration in cell culture media MAbs 2011 3 577 583 22123059 10.4161/mabs.3.6.17959 PMC3242844

[B8] Johnson KA Paisley-Flango K Tangarone BS Porter TJ Rouse JC Cation exchange-HPLC and mass spectrometry reveal C-terminal amidation of an IgG1 heavy chain Anal Biochem 2007 360 75 83 17113563 10.1016/j.ab.2006.10.012

[B9] Cuttitta F Peptide amidation: signature of bioactivity Anat Rec 1993 87 93 172–173; discussion 193–175 8389534 10.1002/ar.1092360112

[B10] Merkler DJ C-terminal amidated peptides: production by the in vitro enzymatic amidation of glycine-extended peptides and the importance of the amide to bioactivity Enzyme Microb Technol 1994 16 450 456 7764886 10.1016/0141-0229(94)90014-0

[B11] In Y Fujii M Sasada Y Ishida T Structural studies on C-amidated amino acids and peptides: structures of hydrochloride salts of C-amidated Ile, Val, Thr, Ser, Met, Trp, Gln and Arg, and comparison with their C-unamidated counterparts Acta Crystallogr B 2001 57 72 81 11173369 10.1107/s0108768100013975

[B12] Bolkenius FN Ganzhorn AJ Peptidylglycine alpha-amidating mono-oxygenase: Neuropeptide amidation as a target for drug design Gen Pharmacol 1998 31 655 659 9809459 10.1016/s0306-3623(98)00192-x

[B13] Prigge ST Mains RE Eipper BA Amzel LM New insights into copper monooxygenases and peptide amidation: structure, mechanism and function Cell Mol Life Sci 2000 57 1236 1259 11028916 10.1007/PL00000763 PMC11146793

[B14] Hayashi N Kayo T Sugano K Takeuchi T Production of bioactive gastrin from the non-endocrine cell lines CHO and COS-7 FEBS Lett 1994 337 27 32 8276107 10.1016/0014-5793(94)80623-3

[B15] Wu SC RNA interference technology to improve recombinant protein production in Chinese hamster ovary cells Biotechnol Adv 2009 27 417 422 19289164 10.1016/j.biotechadv.2009.03.002

[B16] Amarzguioui M Rossi JJ Kim D Approaches for chemically synthesized siRNA and vector-mediated RNAi FEBS Lett 2005 579 5974 5981 16199038 10.1016/j.febslet.2005.08.070

[B17] Santiago Y Chan E Liu PQ Orlando S Zhang L Urnov FD Holmes MC Guschin D Waite A Miller JC Rebar EJ Gregory PD Klug A Collingwood TN Targeted gene knockout in mammalian cells by using engineered zinc-finger nucleases Proc Natl Acad Sci U S A 2008 105 5809 5814 18359850 10.1073/pnas.0800940105 PMC2299223

[B18] Urnov FD Rebar EJ Holmes MC Zhang HS Gregory PD Genome editing with engineered zinc finger nucleases Nat Rev Genet 2010 11 636 646 20717154 10.1038/nrg2842

[B19] Klug A The discovery of zinc fingers and their applications in gene regulation and genome manipulation Annu Rev Biochem 2010 79 213 231 20192761 10.1146/annurev-biochem-010909-095056

[B20] Lim SF Chuan KH Liu S Loh SO Chung BY Ong CC Song Z RNAi suppression of Bax and Bak enhances viability in fed-batch cultures of CHO cells Metab Eng 2006 8 509 522 16860584 10.1016/j.ymben.2006.05.005

[B21] Sung YH Hwang SJ Lee GM Influence of down-regulation of caspase-3 by siRNAs on sodium-butyrate-induced apoptotic cell death of Chinese hamster ovary cells producing thrombopoietin Metab Eng 2005 7 457 466 16169764 10.1016/j.ymben.2005.08.001

[B22] Sung YH Lee JS Park SH Koo J Lee GM Influence of co-down-regulation of caspase-3 and caspase-7 by siRNAs on sodium butyrate-induced apoptotic cell death of Chinese hamster ovary cells producing thrombopoietin Metab Eng 2007 9 452 464 17892962 10.1016/j.ymben.2007.08.001

[B23] Wong DC Wong KT Nissom PM Heng CK Yap MG Targeting early apoptotic genes in batch and fed-batch CHO cell cultures Biotechnol Bioeng 2006 95 350 361 16894638 10.1002/bit.20871

[B24] Ngantung FA Miller PG Brushett FR Tang GL Wang DI RNA interference of sialidase improves glycoprotein sialic acid content consistency Biotechnol Bioeng 2006 95 106 119 16673415 10.1002/bit.20997

[B25] Mori K Kuni-Kamochi R Yamane-Ohnuki N Wakitani M Yamano K Imai H Kanda Y Niwa R Iida S Uchida K Shitara K Satoh M Engineering Chinese hamster ovary cells to maximize effector function of produced antibodies using FUT8 siRNA Biotechnol Bioeng 2004 88 901 908 15515168 10.1002/bit.20326

[B26] Imai-Nishiya H Mori K Inoue M Wakitani M Iida S Shitara K Satoh M Double knockdown of alpha1,6-fucosyltransferase (FUT8) and GDP-mannose 4,6-dehydratase (GMD) in antibody-producing cells: a new strategy for generating fully non-fucosylated therapeutic antibodies with enhanced ADCC BMC Biotechnol 2007 7 84 18047682 10.1186/1472-6750-7-84 PMC2216013

[B27] Kim SH Lee GM Down-regulation of lactate dehydrogenase-A by siRNAs for reduced lactic acid formation of Chinese hamster ovary cells producing thrombopoietin Appl Microbiol Biotechnol 2007 74 152 159 17086415 10.1007/s00253-006-0654-5

[B28] Hong WW Wu SC A novel RNA silencing vector to improve antigen expression and stability in Chinese hamster ovary cells Vaccine 2007 25 4103 4111 17428585 10.1016/j.vaccine.2007.02.012

[B29] Wu SC Hong WW Liu JH Short hairpin RNA targeted to dihydrofolate reductase enhances the immunoglobulin G expression in gene-amplified stable Chinese hamster ovary cells Vaccine 2008 26 4969 4974 18602963 10.1016/j.vaccine.2008.06.081

[B30] Liu PQ Chan EM Cost GJ Zhang L Wang J Miller JC Guschin DY Reik A Holmes MC Mott JE Collingwood TN Gregory PD Generation of a Triple-Gene Knockout Mammalian Cell Line Using Engineered Zinc-Finger Nucleases Biotechnol Bioeng 2010 106 97 105 20047187 10.1002/bit.22654

[B31] Cost GJ Freyvert Y Vafiadis A Santiago Y Miller JC Rebar E Collingwood TN Snowden A Gregory PD BAK and BAX Deletion Using Zinc-Finger Nucleases Yields Apoptosis-Resistant CHO Cells Biotechnol Bioeng 2010 105 330 340 19777580 10.1002/bit.22541

[B32] Yamane-Ohnuki N Kinoshita S Inoue-Urakubo M Kusunoki M Iida S Nakano R Wakitani M Niwa R Sakurada M Uchida K Shitara K Satoh M Establishment of FUT8 knockout Chinese hamster ovary cells: an ideal host cell line for producing completely defucosylated antibodies with enhanced antibody-dependent cellular cytotoxicity Biotechnol Bioeng 2004 87 614 622 15352059 10.1002/bit.20151

[B33] Malphettes L Freyvert Y Chang J Liu PQ Chan E Miller JC Zhou Z Nguyen T Tsai C Snowden AW Collingwood TN Gregory PD Cost GJ Highly Efficient Deletion of FUT8 in CHO Cell Lines Using Zinc-Finger Nucleases Yields Cells That Produce Completely Nonfucosylated Antibodies Biotechnol Bioeng 2010 106 774 783 20564614 10.1002/bit.22751

[B34] Klug A The discovery of zinc fingers and their development for practical applications in gene regulation and genome manipulation Q Rev Biophys 2010 43 1 21 20478078 10.1017/S0033583510000089

[B35] Durai S Mani M Kandavelou K Wu J Porteus MH Chandrasegaran S Zinc finger nucleases: custom-designed molecular scissors for genome engineering of plant and mammalian cells Nucleic Acids Res 2005 33 5978 5990 16251401 10.1093/nar/gki912 PMC1270952

